# Unveiling the hidden risk of caspofungin: insights from three adverse event reporting systems and network pharmacology integration

**DOI:** 10.3389/fphar.2025.1632488

**Published:** 2025-08-13

**Authors:** Zhengfu Li, Zhiwei Cui, De Xie, Fan Zou, Chengyu Zhu

**Affiliations:** ^1^ Department of Respiratory and Critical Care Medicine, Affiliated Hospital of Zunyi Medical University, Zunyi, China; ^2^ Department of Obstetrics and Gynecology, the First Affiliated Hospital of Xi’an Jiaotong University, Xi’an, China; ^3^ Department of Internal Medicine, Xiang’an Hospital of Xiamen University, School of Medicine, Xiamen University, Xiamen, China

**Keywords:** caspofungin, adverse drug events, drug-induced liver injury, real-world data, PI3K/AKT signaling

## Abstract

**Background:**

Caspofungin, the first FDA-approved echinocandin antifungal agent, plays a vital role in managing invasive fungal infections (IFIs). Despite its established efficacy, large-scale real-world safety evaluations remain limited. This study provides a comprehensive pharmacovigilance analysis of caspofungin’s safety profile.

**Methods:**

Adverse drug events (ADEs) associated with caspofungin were extracted from the FDA Adverse Event Reporting System (FAERS), the Japanese Adverse Drug Event Reporting Database and the Canadian Vigilance Adverse Reaction Database (CVARD) databases. Signal detection utilized four methods: reporting odds ratio proportional reporting ratio Bayesian confidence propagation neural network and multiple gamma-Poisson shrinkage Time-to-onset (TTO) analysis was conducted using FDA Adverse Event Reporting System data, and network pharmacology approaches were employed to investigate potential molecular mechanisms, particularly in caspofungin-related liver injury.

**Results:**

A total of 2,270, 161, and 128 ADE reports were retrieved from FAERS, JADER, and CVARD, respectively. “Hepatobiliary disorders” and “infections and infestations” are overlapping positive signals from three databases at the system organ class level. ADEs such as hypokalemia, sepsis, and drug ineffectiveness were consistent with the drug label. Unexpected signals included prolonged QT interval, cardiac arrest, septic shock, and cholestasis. Cross-database overlap included “drug ineffective” and “toxic skin eruption” between FAERS and JADER, and “renal failure,” “photodermatitis” between FAERS and CVARD. TTO analysis revealed that 89.95% of ADEs occurred within the first month, with a median onset time of 6 days. Network pharmacology identified PI3K/Akt and HIF-1 pathways as mechanisms underlying caspofungin-induced liver injury.

**Conclusion:**

This study highlights both expected and unexpected ADEs of caspofungin, emphasizing the importance of clinical vigilance and molecular research to enhance patient safety and therapeutic outcomes.

## 1 Introduction

Various fungal species are integral components of the human microbiota, inhabiting multiple anatomical sites, including the skin, respiratory system, genitourinary tract, oral cavity, and gastrointestinal tract. These fungi play crucial roles in maintaining host health by contributing to physiological balance and immune homeostasis (Zhai et al., 2020). However, in individuals with compromised immune systems, commensal fungi can transition into invasive pathogens, disseminating systemically and causing invasive fungal infections (IFIs) that affect multiple organs and systems (Friedman and Schwartz, 2023). IFIs represent a significant clinical challenge, particularly in critically ill patients or those with immunosuppression due to underlying diseases or immunosuppressive therapies. Fungal infections are a major global health concern, contributing to an estimated 1.5 million deaths and 1.7 billion superficial infections annually, imposing a considerable economic burden on healthcare systems (Arastehfar et al., 2020).

Caspofungin, a semisynthetic derivative of pneumocandin B, was approved by the U.S. Food and Drug Administration (FDA) in 2001 as the first member of the echinocandin class to combat fungal infections. Its mechanism of action involves the inhibition of β-(1,3)-D-glucan synthase, a critical enzyme in fungal cell wall biosynthesis, leading to suppression of β-(1,3)-D-glucan synthesis and subsequent fungal cell wall disruption. A large number of randomized controlled trials have shown that caspofungin is an effective option for the treatment of IFIs and is especially suitable as a first-line antifungal agent. Pooled analyses of the phase 2 STRIVE and phase 3 ReSTORE trials, conducted by George R. Thompson and colleagues, showed that mycological eradication was achieved in 100 (65%) of 155 caspofungin-treated patients by day 5, suggesting a potential early treatment advantage (Thompson et al., 2024). In a double-blind trial involving 239 patients with candidemia, caspofungin demonstrated successful outcomes in 73.4% of cases, which was comparable to the 71.7% favorable response rate observed with amphotericin B (Mora-Duarte et al., 2002).

With the increasing clinical use of caspofungin, reports of associated ADEs have gradually risen. The most common treatment-related ADEs that occurred in at least 5% of patients in the caspofungin group in the ReSTORE clinical trial included pyrexia, hypokalemia, pneumonia, septic shock, and anemia (Thompson et al., 2023). However, the long-term efficacyand safety profile of caspofungin has been primarily documented through clinical trials, which are often limited by small sample sizes and stringent inclusion criteria in premarketing studies. To address these limitations and better assess the real-world safety of caspofungin, this pharmacovigilance analysis was conducted, driven by its extensive clinical utilization and the need for comprehensive adverse event evaluations.

In recent years, three major real-world spontaneous adverse event reporting systems—the FDA Adverse Event Reporting System (FAERS), the Japanese Adverse Drug Event Report (JADER), and the Canada Vigilance Adverse Reaction Database (CVARD)—have collected substantial data on adverse drug events (ADEs) across diverse patient populations. The objective of this study was to assess the safety profile of caspofungin by analyzing post-marketing ADE reports from these three databases. The primary goal of these findings is to offer valuable insights for clinical monitoring, enhance early detection of potential safety signals, and support the identification of hazards associated with caspofungin therapy.

## 2 Methods

### 2.1 Data collecting and processing

**FIGURE 1:**
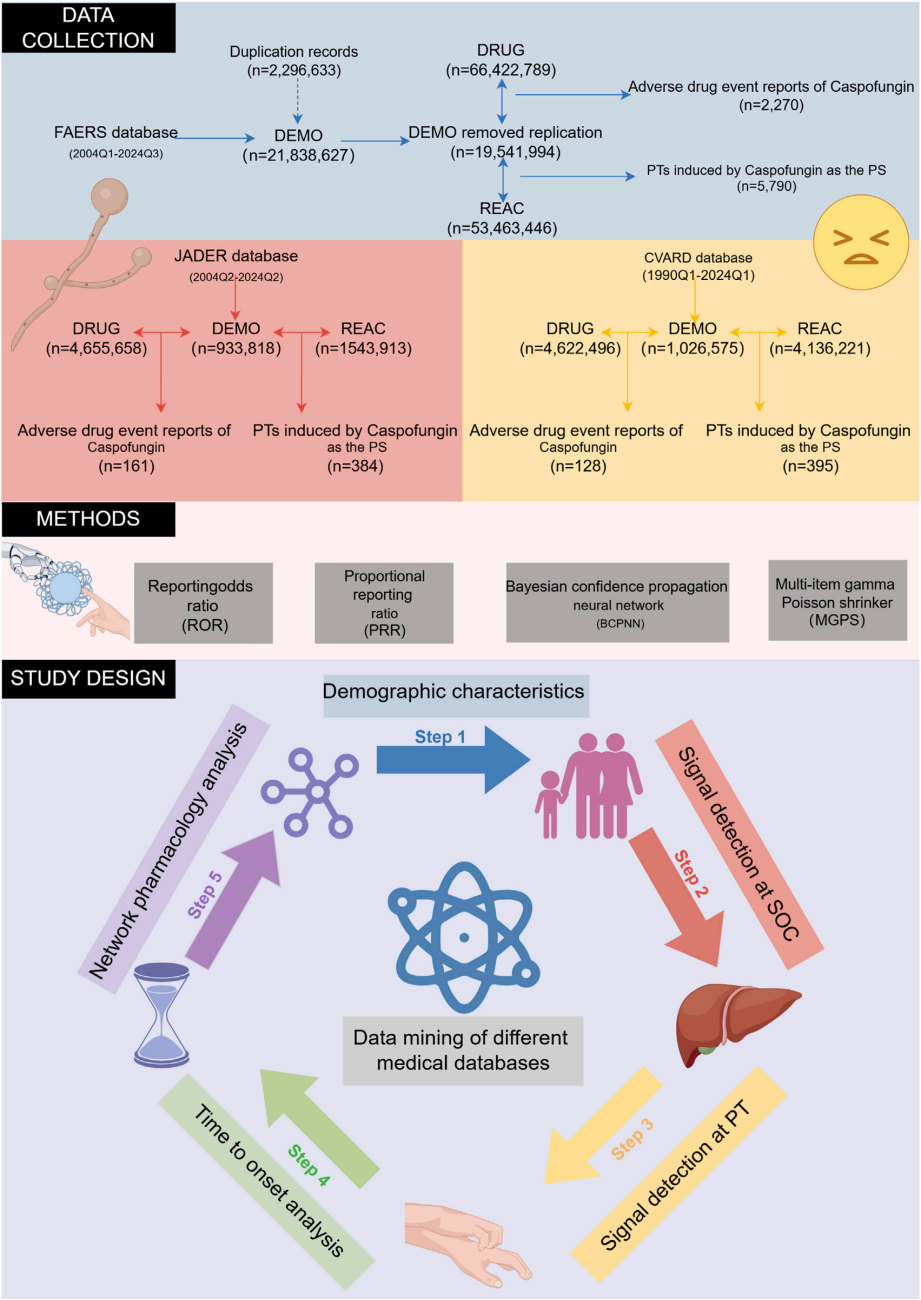
The flowchart of the entire study is outlined. The study is structured into three main phases: data collection and preprocessing, application of disproportionality analysis methods, and the key components of the study. FAERS, FDA Adverse Event Reporting System; JADER, Japanese Adverse Drug Event Report; CVARD, Canada Vigilance Adverse Reaction Database; PT, preferred term; SOC, system organ class.

The FAERS database, the largest repository of ADE reports, contains data from healthcare professionals, manufacturers, and patients since 2004. Using both the generic (CASPOFUNGIN) and trade (CANCIDAS) names, ADEs associated with caspofungin from Q1 2004 to Q3 2024 were analyzed. To address non-standardized drug names and potential duplicates, FDA-recommended deduplication procedures were applied. Only reports listing caspofungin as the primary suspect (PS) drug were included. Sensitivity analyses minimized confounding effects of concomitant medications (Liu et al., 2024). Adverse events were standardized using Medical Dictionary for Regulatory Activities (MedDRA, version 27.0) and categorized by system organ class (SOC). After cleaning, 2,270 reports identified caspofungin as the PS drug, with 5,790 preferred terms (PTs) associated (Figure 1).

The JADER database compiles ADE reports submitted to the Pharmaceuticals and Medical Devices Agency (PMDA) by healthcare professionals, marketing authorization holders, patients, and consumers since 2004 (Oura et al., 2024). All data are anonymized and curated by Japanese regulatory authorities. For this study, only records where caspofungin was listed as a “suspected drug” were analyzed. Caspofungin was identified in the JADER database using its Japanese name, “カスポファンギン酢酸塩,” covering data from Q2 2004 to Q2 2024. After data cleaning, a total of 161 ADE reports identified caspofungin as the suspected drug, with 384 PTs associated (Figure 1).

The Canada Vigilance Adverse Reaction Database (CVARD), established in 1965, collects reports of suspected adverse reactions submitted by healthcare professionals, consumers, and manufacturers (Ali et al., 2015). For this study, data from Q1 1990 to Q1 2024 were extracted. The various datasets were merged using “Report_ID” as a unique identifier, and duplicate records were removed to ensure data integrity (Maity and Longo, 2021). As with JADER, only ADE reports where caspofungin was listed as the suspected drug were included in the analysis. To ensure consistency and comparability across all analyzed databases, adverse events were harmonized using the PTs from the MedDRA version 27.0.

### 2.2 Signal detection

Disproportionality analysis is a widely used data mining technique to assess whether suspected drug-induced events are being reported more frequently than expected for the target drug. The primary objective is to identify statistically significant drug-ADE associations and estimate their effect sizes. To enhance the robustness and reliability of the results, we utilized a combination of disproportionality analysis methods, including two frequency-based approaches—reporting odds ratio (ROR) and proportional reporting ratio (PRR)—as well as two Bayesian approaches—Bayesian confidence propagation neural network (BCPNN) and multi-item gamma Poisson shrinker (MGPS) (van Puijenbroek et al., 2002). By combining these complementary approaches, we aimed to enhance the accuracy and reliability of signal detection. Further details on the formulas and thresholds for each method are provided in Table 1. At the SOC level, any SOC meeting the algorithm threshold is marked as a positive SOC. Furthermore, SOCs that meet the thresholds of all four algorithms are considered significant SOCs. At the PT level, drug-ADE pairs meeting all four algorithm thresholds were defined as positive signals. Modified relative risk (RR) was the primary statistic used to assess drug-ADE associations. To account for the potential inflation of false positives due to multiple comparisons, *P*-values were adjusted using the Bonferroni correction. Signals not included in the drug’s official labeling were categorized as unexpected. All data processing and analysis were conducted using Microsoft Excel 2019 and R software (version 4.2.1).

**TABLE 1:** A two-by-two contingency table and detailed formulas for disproportionality analysis.

Drug category	Target adverse drug event	Other adverse drug events	Sums
Caspofungin	a	b	a+b
Other drugs (Non-caspofungin)	c	d	c + d
Total	a+c	b + d	a+b + c + d

Methods	Formula	Threshold
ROR	$ROR=\frac{a/c}{b/d}$	a≥3
	$SE\left(lnROR\right)=\sqrt{\left(\frac{1}{a}+\frac{1}{b}+\frac{1}{c}+\frac{1}{d}\right)}$	
	$95\%\text{CI}=e^{\ln\left(\text{ROR}\right)\pm 1.96\text{se}}$	$95\%\text{CI}\left(\text{lower limit}\right)>1$
PRR	$\text{PRR}=\frac{a/\left(a+b\right)}{c/\left(c+d\right)}$	PRR> 2
	χ^2^ = [(ad-bc)^2^](a+b + c + d)/[(a+b) (c + d) (a+c) (b + d)]	χ2≥4
BCPNN	$\text{IC}=\log_{2}\frac{p\left(x,y\right)}{p\left(x\right)p\left(y\right)}=\log_{2}\frac{a\left(a+b+c+d\right)}{\left(a+b\right)\left(a+c\right)}$	IC025>0
	$E\left(\text{IC}\right)=\log_{2}\frac{\left(a+\gamma 11\right)\left(a+b+c+d+\alpha \right)\left(a+b+c+d+\beta \right)}{\left(a+b+c+d+\gamma \right)\left(a+b+\alpha 1\right)\left(a+c+\beta 1\right)}$	
	$V\left(\text{IC}\right)=\frac{1}{{\left(\ln2\right)}^{2}}\left[\frac{\left(a+b+c+d\right)-a+\gamma -\gamma 11}{\left(a+\gamma 11\right)\left(1+a+b+c+d+\gamma \right)}+\frac{\left(a+b+c+d\right)-\left(a+b\right)+a-\alpha 1}{\left(a+b+\alpha 1\right)\left(1+a+b+c+d+\alpha \right)}+\frac{\left(a+b+c+d+\alpha \right)-\left(a+c\right)+\beta -\beta 1}{\left(a+b+\beta 1\right)\left(1+a+b+c+d+\beta \right)}\right]$	
	$\gamma =\gamma 11\frac{\left(a+b+c+d+\alpha \right)\left(a+b+c+d+\beta \right)}{\left(a+b+\alpha 1\right)\left(a+c+\beta 1\right)}$	
	$\text{IC}-2\text{SD}=E\left(\text{IC}\right)-2\sqrt{V\left(\text{IC}\right)}$	
EBGM	$\text{EBGM}=\frac{a\left(a+b+c+d\right)}{\left(a+c\right)\left(a+b\right)}$	EBGM 05>2
	$\text{SE}\left(\text{lnEBGM}\right)=\sqrt{\left(\frac{1}{a}+\frac{1}{b}+\frac{1}{c}+\frac{1}{d}\right)}$	
	$95\%\text{CI}=e^{\ln\left(\text{EBGM}\right)\pm 1.96\text{se}}$	

The methods and thresholds for ROR, PRR, BCPNN, and EBGM are described as follows. a, the number of reports containing both caspofungin and target adverse events; b, the number of reports involving other adverse events with caspofungin; c, the number of reports documenting target adverse events with other drugs; d, the number of reports involving other drugs and non-target adverse events. ROR, reporting odds ratio; PRR, proportional reporting ratio; BCPNN, Bayesian confidence propagation neural network; EBGM, empirical Bayesian geometric mean; 95% CI, 95% confidence interval; χ^2^: chi-squared; IC, information component; IC025, Information Component 2.fifth percentile; E (IC), expected IC; V(IC), variance of IC; EBGM05, Empirical Bayes Geometric Mean fifth percentile.

### 2.3 Time to onset analysis

Time-to-onset (TTO) analyses were performed using the FAERS database, as JADER and CVARD lacked sufficient data. TTO was defined as the time between the initiation of caspofungin (START_DT) and the adverse event occurrence (EVENT_DT). Reports with incomplete, erroneous, or inconsistent dates were excluded to ensure accuracy. TTO data were summarized using medians, quartiles, and extreme values. The Weibull shape parameter (WSP) test assessed changes in ADE incidence over time. The scale parameter (α) indicated distribution spread, while the shape parameter (β) described the curve form, with β > 1 indicating left skew and β < 1 indicating right skew.

### 2.4 Causality assessment using the Bradford Hill criteria

The Austin Bradford Hill criteria, initially developed to evaluate the causal relationships between environmental factors and disease outcomes, have become widely utilized in epidemiological research and form a foundational framework in pharmacovigilance studies (Chen et al., 2024). In this study, a modified version of the Bradford Hill criteria was applied to assess the potential causal link between caspofungin and drug-induced liver injury (DILI). This framework integrates key aspects of the Bradford Hill criteria and relies on existing literature as well as disproportionality analysis data. The assessment covers multiple dimensions: the strength of the association, analogy, biological plausibility/empirical evidence, specificity, consistency, temporal relationship, reversibility, and coherence.

### 2.5 Caspofungin-related DILI network construction

Using the keyword “caspofungin,” its chemical structure and SMILES notation were retrieved from the PubChem database. SwissTargetPrediction (www.swisstargetprediction.ch) integrates both two-dimensional and three-dimensional molecular similarity approaches, leveraging known bioactive compound data to train its predictive models. It enables the accurate prediction of potential protein targets based on small-molecule structures, offering high reliability in target identification. Developed by the Swiss Institute of Bioinformatics, this platform forms part of their integrated drug design suite, aiming to advance precision in target discovery and facilitate innovative drug development (Daina et al., 2019). The SuperPred (https://prediction.charite.de) employs chemical similarity and the similarity principle to associate drug-like compounds with molecular targets and therapeutic indications. It offers functionalities such as target prediction, drug indication analysis, and confidence assessment, making it well suited for exploring potential targets and indications of novel compounds (Nickel et al., 2014). Based on a previous study, target selection thresholds were set using the results from the SwissTargetPrediction (human, probability >0.01) and SuperPred databases (probability >0.5) (Xie et al., 2025). By further analyzing the SMILES notation of caspofungin, potential drug targets were identified using these two databases. After removing duplicates and standardizing the data *via* UniProt, a total of 152 targets were ultimately identified.

GeneCards (www.genecards.org) is a comprehensive and authoritative compendium of annotated information on human genes. By integrating data on genetic variants, differential expression, and functional annotations, it enables researchers to identify potential disease “candidate targets” with greater precision (Safran et al., 2010). Phenolyzer (https://phenolyzer.wglab.org/) performs intelligent analyses by integrating multidimensional gene–gene relationships, such as protein–protein interactions, shared signaling pathways, and gene families. It demonstrates excellent performance in prioritizing candidate genes for Mendelian and complex diseases, as well as in discovering novel disease–gene associations (Yang et al., 2015). DILI-associated targets were identified using GeneCards (relevance score ≥10) (Wang et al., 2025) and Phenolyzer (relevance score ≥0.50) (Fang et al., 2017). Overlapping targets between caspofungin and DILI were identified using R software and further analyzed with the STRING database to construct protein-protein interaction (PPI) networks. Hub genes were identified using Cytoscape 3.7.1 based on betweenness centrality. Gene Ontology (GO) and KEGG pathway enrichment analyses were conducted on intersecting genes using the clusterProfiler package (v4.4.4) in R to explore biological pathways potentially involved in caspofungin-associated DILI. Figure 1 summarizes the study design, including target prediction workflows, database queries, and disproportionality analysis.

## 3 Results

### 3.1 Descriptive analysis

**FIGURE 2:**
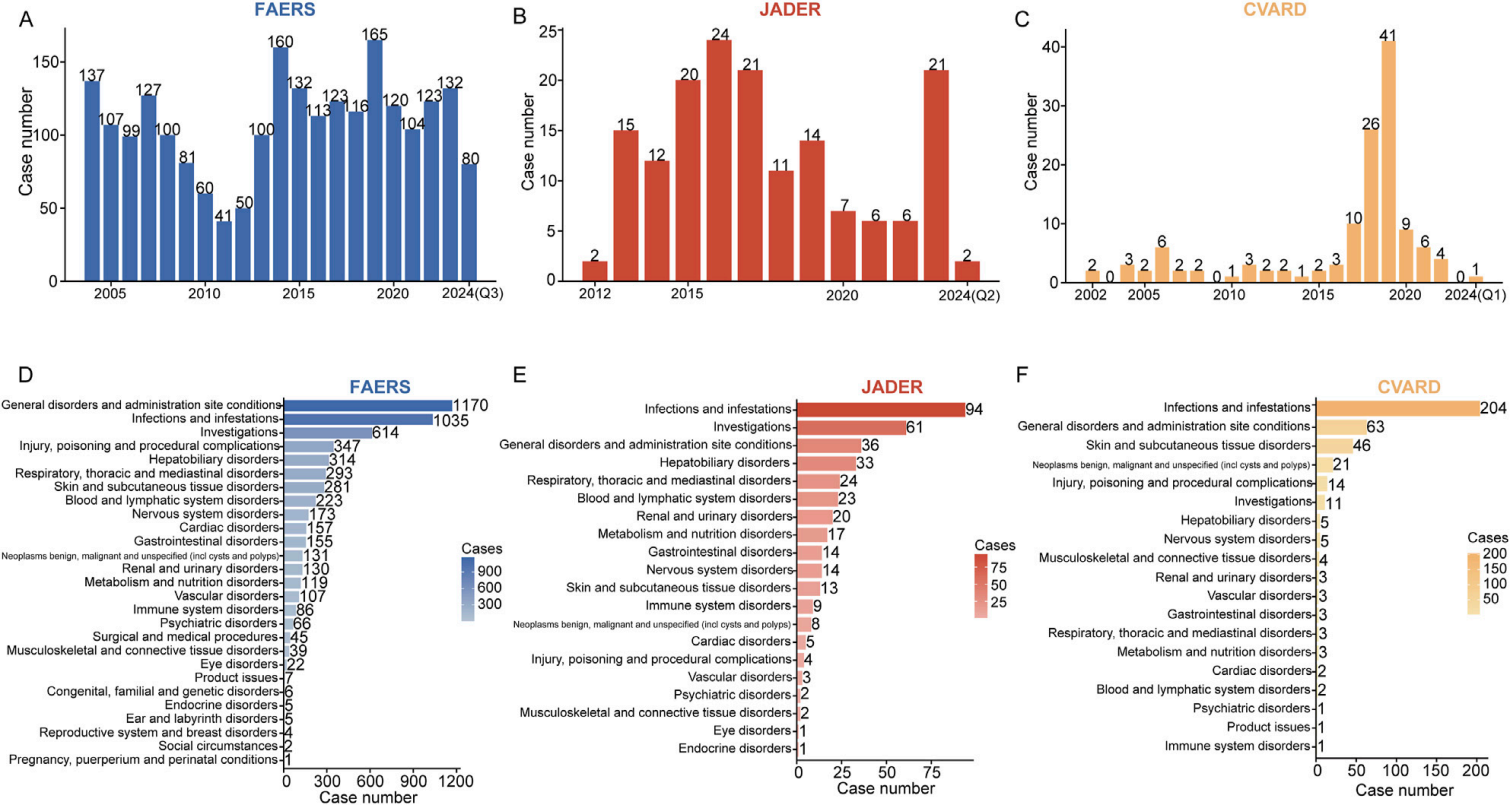
The annual number of adverse drug events (ADEs) reported across different medical databases and their distribution at the system organ class (SOC) level are presented. The bar charts display the number of ADEs reported per year in FAERS **(A)**, JADER **(B)**, and CVARD **(C)**. At the SOC level, ADE reports were counted and sorted in descending order. The ADE report distributions for FAERS **(D)**, JADER **(E)**, and CVARD **(F)** are represented in red, blue, and yellow, respectively. The intensity of the color indicates the volume of reports, with darker shades corresponding to a higher number of reports. Q1, the first quarter; Q2, the second quarter; Q3, the third quarter.

Following deduplication, the number of caspofungin-associated ADEs identified was 2,270 in FAERS, 161 in JADER, and 128 in CVARD. Detailed demographic characteristics of these cases are presented in Tables 2–4. Across all three databases, males consistently outnumbered females: 52.5% vs 35.3% in FAERS (Table 2), 60.9% vs 27.1% in JADER (Table 3), and 66.4% vs 32.0% in CVARD (Table 4). Regarding age distribution, the majority of patients in the FAERS database were between 18 and 65 years (1,077 cases, 44.4%), while the JADER database had the highest proportion in the 15–65 age group (67 cases, 41.6%). In contrast, most cases in CVARD were found in the under-18 age group (76 cases, 59.4%). Weight data were largely unavailable across all databases. Regarding ADE outcomes, death was the most commonly reported consequence: 919 cases (40.5%) in FAERS, 109 cases (28.4%) in JADER, and 30 cases (23.4%) in CVARD. The most frequently reported indication for caspofungin use varied across the databases: fungal infection was the leading indication in FAERS (178 cases, 19.0%), febrile neutropenia in JADER (44 cases, 19.0%), and antifungal prophylaxis in CVARD (78 cases, 56.5%)Figures 2A–C illustrate the annual reports of caspofungin-related ADE in the FAERS, JADER, and CVAR databases, respectively. In the FAERS database, the number of reports consistently exceeded 40, with reports remaining above 100 annually from 2013 to 2023 (Figure 2A). In JADER, reports from 2013 to 2019 consistently numbered no less than 10 annually, maintaining a relatively high level (Figure 2B). In contrast, the CVARD showed relatively low numbers of reports except between 2017 and 2019 (Figure 2C).

**TABLE 2:** Demographic characteristics of ADEs reported in the FAERS database with caspofungin as the primary suspect drug.

Characteristics	Case number	Case proportion, %
Sex, n (%)		
Female	802	35.3%
Male	1,192	52.5%
Unknown	276	12.2%
Age (years)		
<18	254	11.2%
18–65	1,007	44.4%
>65	548	24.1%
Unknown	461	20.3%
Weight (kg)		
<50	182	8.0%
50–100	430	18.9%
>100	32	1.4%
Unknown	1,626	71.6%
Reported Countries (top five)		
France	381	16.8%
United States	364	16.0%
Japan	285	12.6%
China	110	4.8%
Germany	105	4.6%
Reported person		
Health professional	1979	87.2%
Consumer	226	9.9%
Unknown	65	2.9%
Outcome		
HO	489	21.5%
LT	130	5.7%
DS	18	0.8%
RI	6	0.3%
DE	919	40.5%
OT	427	18.8%
Unknown	281	12.4%
Indication (top five)		
Fungal infection	178	7.8%
Systemic *candida*	177	7.8%
Bronchopulmonary aspergillosis	129	5.7%
*Candida* infection	117	5.2%
Antifungal prophylaxis	110	4.8%

FAERS, FDA Adverse Event Reporting System. DE, death; DS, disability; HO, hospitalization-initial or prolonged; LT, life-threatening; OT, other serious; RI, required intervention.

**TABLE 3:** Demographic characteristics of ADEs reported in the JADER database with caspofungin as the suspected drug.

Characteristics	Case number	Case proportion, %
Sex, n (%)		
Female	44	27.1%
Male	98	60.9%
Unknown	19	11.8%
Age (years)		
<15	12	7.4%
15–65	67	41.6%
>65	55	34.2%
Unknown	27	16.8%
Weight (kg)		
<50	32	19.9%
50–100	55	34.2%
>100	0	0.0%
Unknown	74	45.9%
Outcome		
Recovery (recovery but with sequelae)	1	0.3%
Rehabilitation	77	20.1%
Minor rehabilitation	47	12.2%
Death	109	28.4%
Non-rehabilitated	38	9.9%
Missing	112	29.2%
Indication (top five)		
Febrile neutropenia	44	19.0%
Systemic *candida*	22	9.5%
Bronchopulmonary aspergillosis	15	6.4%
Antifungal prophylaxis	14	6.0%
Fungal infection	11	4.7%

JADER, Japanese Adverse Drug Event Report.

**TABLE 4:** Demographic characteristics of ADEs reported in the CVARD with caspofungin as the suspected drug.

Characteristics	Case number	Case proportion, %
Sex, n (%)		
Female	41	32.0%
Male	85	66.4%
Unknown	2	1.6%
Age (years)		
<18	76	59.4%
18–65	18	14.1%
>65	7	5.5%
Unknown	27	21.1%
Weight (kg)		
50–100	4	3.1%
>100	1	0.8%
Unknown	123	96.1%
Reported person		
Health professional	108	84.4%
Consumer	5	3.9%
Unknown	15	11.7%
Outcome		
Death	30	23.4%
Hospitalization-required	12	9.4%
Not serious	4	3.1%
Other	82	64.1%
Indication (top five)		
Antifungal prophylaxis	78	56.5%
Fungal infection	12	8.6%
Systemic *candida*	6	4.3%
Pneumonia	3	2.1%
Acute Lymphocytic leukaemia	2	1.4%

CVARD, Canada Vigilance Adverse Reaction Database.

### 3.2 Signal detection at the SOC level

The signal intensities and the reported cases for caspofungin at the SOC level across the three databases are summarized in Supplementary Table S1. SOCs are ranked in descending order of reported cases. Caspofungin-related ADEs were associated with 25 SOCs in FAERS, 16 in JADER, and 14 in CVARD. In FAERS, the leading SOCs included general disorders and administration site conditions (1,170 cases), infections and infestations (1,035 cases), investigations (614 cases), injury, poisoning, and procedural complications (347 cases), and hepatobiliary disorders (314 cases) (Figure 2D). In JADER, the most frequent SOCs were infections and infestations (94 cases), investigations (61 cases), general disorders and administration site conditions (36 cases), hepatobiliary disorders (33 cases), and respiratory, thoracic, and mediastinal disorders (24 cases) (Figure 2E). In CVARD, the top SOCs were infections and infestations (204 cases), general disorders and administration site conditions (63 cases), skin and subcutaneous tissue disorders (46 cases), neoplasms (benign, malignant, and unspecified) (21 cases), and injury, poisoning, and procedural complications (14 cases) (Figure 2F).

**FIGURE 3:**
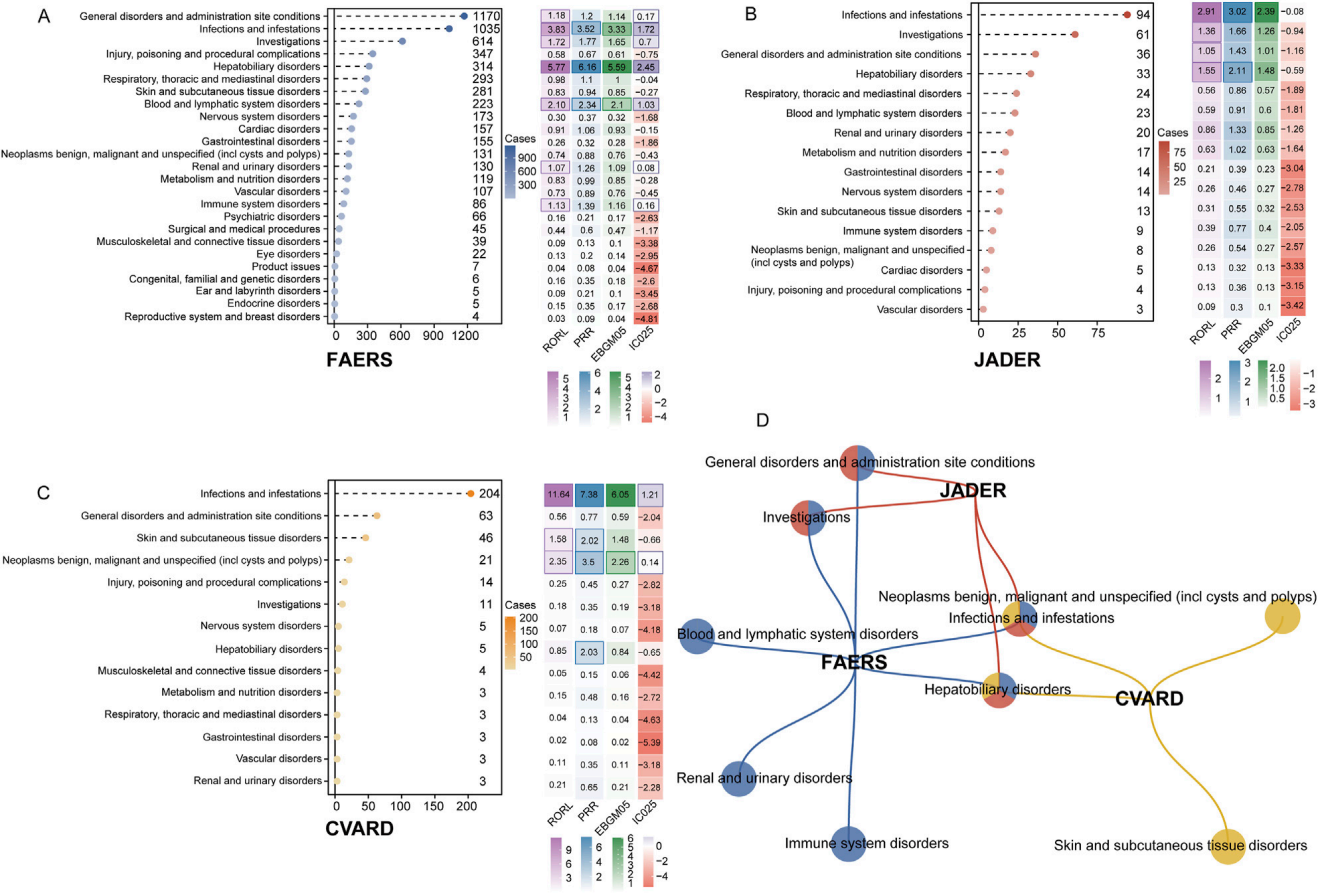
Signal detection at the SOC level is presented. The lollipop chart on the left illustrates the number of adverse event reports associated with each SOC. The heatmap on the right displays the values for RORL, PRR, EBGM05, and IC025. Positive results that met the algorithm thresholds are highlighted with dark-colored borders in the heatmap. The results for FAERS, JADER, and CVARD are shown in **(A–C)**, respectively. **(D)** A network Venn diagrams illustrating the overlap of positive SOCs across the three databases. RORL, lower bound of reporting odds ratio; PRR, proportional reporting ratio; EBGM05, Empirical Bayes Geometric Mean fifth percentile; IC025, Information Component 2.fifth percentile.

After calculating signal values using four disproportionality analysis methods, positive signals were identified at the SOC level across the three databases (Figures 3A–C). In the FAERS database, the most significant SOCs (meeting thresholds for all four disproportionality algorithms) included infections and infestations [1,035 cases; ROR 4.1 (95% CI 3.83–4.39), PRR 3.52, EBGM05 3.33, IC025 1.72], hepatobiliary disorders [314 cases; ROR 6.47 (95% CI 5.77–7.25), PRR 6.16, EBGM05 5.59, IC025 2.45], and blood and lymphatic system disorders [223 cases; ROR 2.4 (95% CI 2.1–2.75), PRR 2.34, EBGM05 2.1, IC025 1.03] (Figure 3A). Other positive SOCs that met at least one algorithm-positive criterion include general disorders and administration site conditions (1,170 cases; ROR 1.26 [95% CI 1.18–1.34], PRR 1.2, EBGM05 1.14, IC025 0.17), renal and urinary disorders (130 cases; ROR 1.27 [95% CI 1.07–1.51], PRR 1.26, EBGM05 1.09, IC025 0.08), and immune system disorders (86 cases; ROR 1.39 [95% CI 1.13–1.72], PRR 1.39, EBGM05 1.39, IC025 0.16). In the JADER database, four positive SOCs were identified: infections and infestations (94 cases; ROR 3.68 [95% CI 2.91–4.64], PRR 3.02, EBGM05 2.39, IC025–0.08), investigations (61 cases; ROR 1.78 [95% CI 1.36–2.35], PRR 1.66, EBGM05 1.26, IC025–0.94), general disorders and administration site conditions (36 cases; ROR 1.47 [95% CI 1.05–2.08], PRR 1.43, EBGM05 1.01, IC025–1.16), and hepatobiliary disorders (33 cases; ROR 2.22 [95% CI 1.55–3.17], PRR 2.11, EBGM05 1.48, IC025–0.59) (Figure 3B). In the CVARD, significant SOCs included infections and infestations (204 cases; ROR 14.19 [95% CI 11.64–17.28], PRR 7.38, EBGM05 6.05, IC025 2.88) and neoplasms benign, malignant, and unspecified (21 cases; ROR 3.64 [95% CI 2.35–5.66], PRR 3.5, EBGM05 2.26, IC025 0.14). Other positive SOCs included skin and subcutaneous tissue disorders (46 cases; ROR 2.15 [95% CI 1.58–2.93], PRR 2.02, EBGM05 1.48, IC025–0.66) and hepatobiliary disorders (5 cases; ROR 2.04 [95% CI 0.85–4.94], PRR 2.03, EBGM05 0.84, IC025–0.65) (Figure 3C). Several SOCs were identified as positive across all three databases, including hepatobiliary disorders and infections and infestations (Figure 3D).

### 3.3 Signal detection at the PT level

After screening and excluding signals unrelated to drug therapy, those influenced by primary pathology, or those representing potential indications, a total of 153 positive PTs were identified in FAERS, 14 in JADER, and 11 in CVARD. These PTs were prioritized based on the number of reported cases in each database. The detailed results are presented in Supplementary Tables S2–S4.

The three most frequently reported positive PTs in the FAERS database were drug ineffective (504 cases; ROR 4.47, PRR 4.16, EBGM05 3.85, IC025 1.92), product use in unapproved indication (124 cases; ROR 6.27, PRR 6.16, EBGM05 5.3, IC025 2.36), septic shock (81 cases; ROR 21.18, PRR 20.89, EBGM05 17.35, IC025 4.06). Additionally, several unexpected signals were identified, including cholestasis (41 cases; ROR 23.81, PRR 23.64, EBGM05 18.23, IC025 4.11), cardio-respiratory arrest (13 cases; ROR 3.27, PRR 3.26, EBGM05 2.07, IC025 0.93), electrocardiogram QT prolongation (13 cases; ROR 3.96, PRR 3.95, EBGM05 2.51, IC025 1.21), and anaphylactic shock (9 cases; ROR 3.95, PRR 3.94, EBGM05 2.28, IC025 1.06) (Supplementary Table S2).

In the JADER database, several positive signals were consistent with those listed in the drug label, including hyperkalemia (15 cases; ROR 15.02, PRR 14.47, EBGM05 8.60, IC025 2.18), blood lactate dehydrogenase increased (7 cases; ROR 26.92, PRR 26.44, EBGM05 12.41, IC025 3.04), and blood alkaline phosphatase increased (5 cases; ROR 18.55, PRR 18.32, EBGM05 7.53, IC025 2.51). Furthermore, eight positive PTs were not explicitly mentioned in the drug label, including sepsis, multiple organ dysfunction syndrome, toxic skin eruption, hypogammaglobulinemia, and staphylococcal infection (Supplementary Table S3).

In the CVARD, signals consistent with the drug label included systemic mycosis (52 cases; ROR 24115.45, PRR 20940.89, EBGM05 4308.81, IC025 11.07) and renal failure (3 cases; ROR 7.59, PRR 7.54, EBGM05 2.42, IC025 1.24). In contrast, nine unexpected signals were identified, including squamous cell carcinoma of the skin, photosensitivity reaction, actinic keratosis, dysplastic naevus, and photodermatitis, among others (Supplementary Table S4).

**FIGURE 4:**
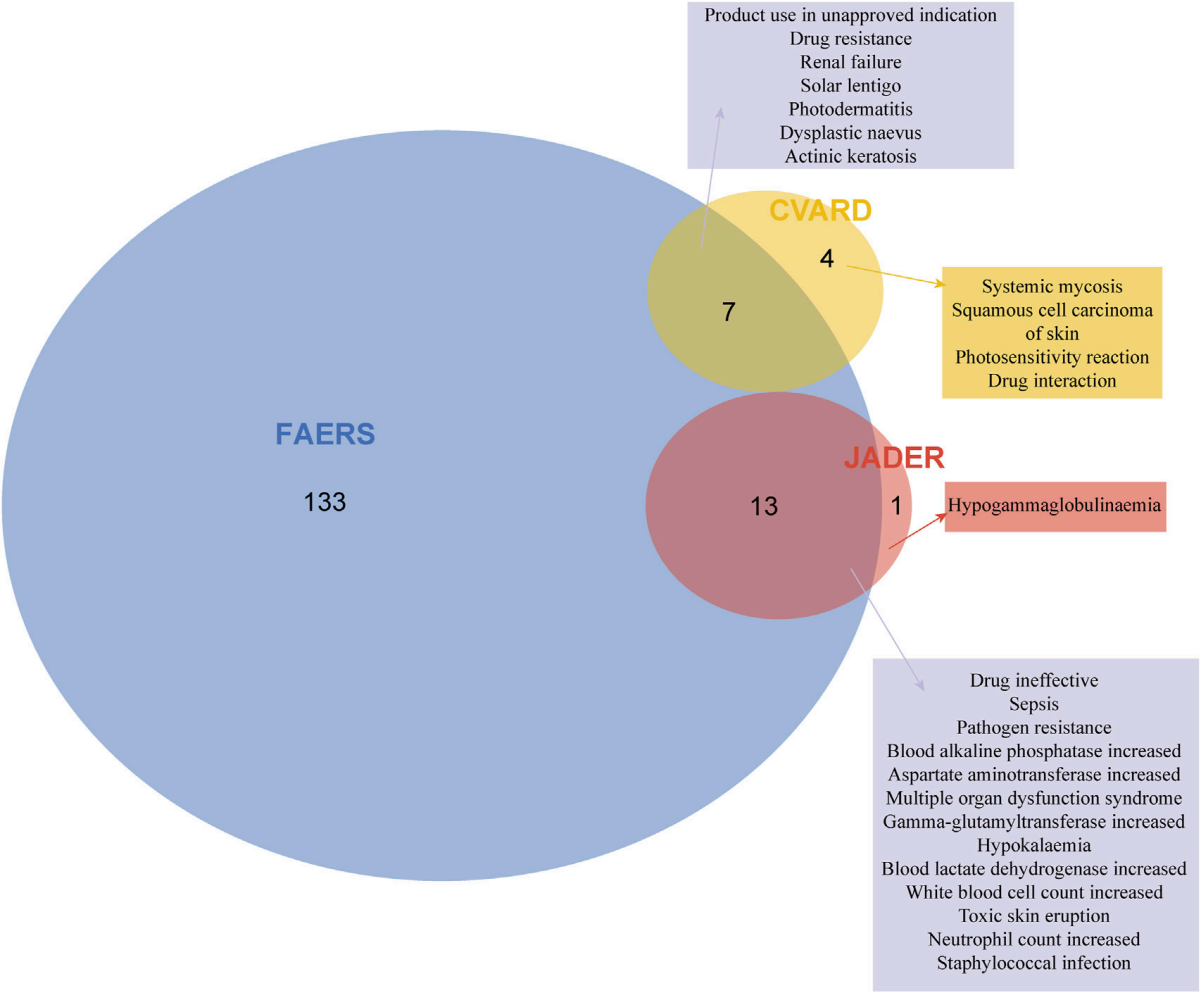
Signal detection at the PT level is presented. After applying the positivity thresholds of the four algorithms and adjusting for Bonferroni’s multiple testing correction, 153, 14, and 11 positive signals were identified in FAERS, JADER, and CVARD, respectively. Venn diagrams illustrate the overlap of positive PTs across the three databases. PT, preferred term.

Notably, ADEs associated with caspofungin at the PT level showed overlap across the three databases. Figure 4 illustrates this convergence of positive signals, revealing a total of 20 overlapping signals. Seven overlapping signals were identified between FAERS and CVARD, including renal failure, solar lentigo, and photodermatitis, among others. Additionally, thirteen overlapping signals were detected between FAERS and JADER, including sepsis, increased blood alkaline phosphatase, increased aspartate aminotransferase, hypokalemia, and toxic skin eruption.

### 3.4 Bradford Hill causality assessment

For caspofungin-associated DILI, most of the Bradford Hill causality criteria were met, including strength of the association, analogy, consistency, temporal relationship, and coherence, suggesting a potential causal link between caspofungin use and the occurrence of DILI. However, limitations in biological plausibility and the database structure precluded a full assessment of specificity and reversibility, and a definitive causal relationship could not be established (see Supplementary Table S5).

### 3.5 The TTO of ADEs associated with caspofungin

**FIGURE 5:**
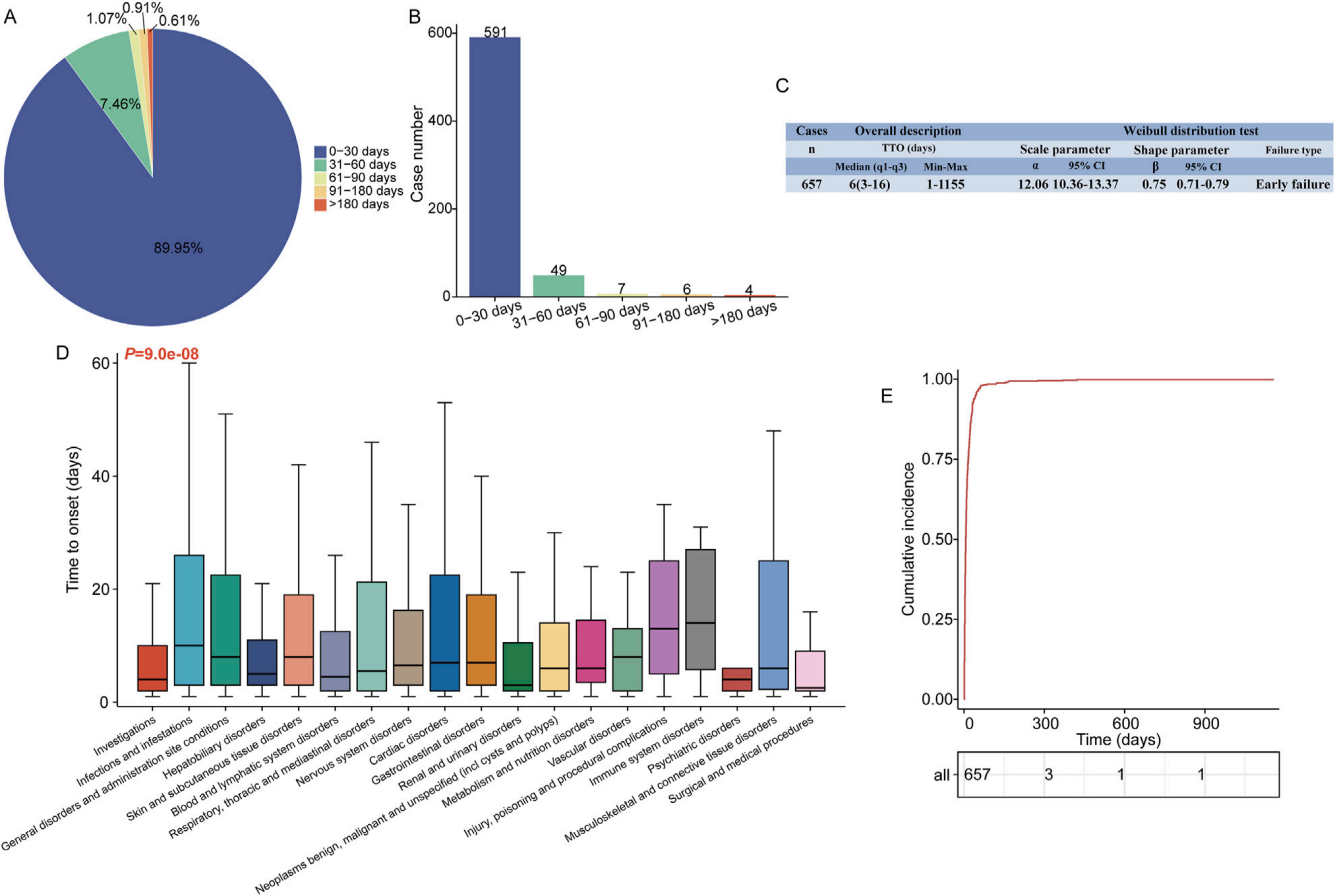
Time to onset analysis (counted in days) of caspofungin-related ADEs at the overall level. The fan **(A)** and frequency bar **(B)** charts show the percentage and number of TTO reports across various time periods, respectively. **(C)** Overall description of the TTO report (including median and extreme values) and the results of the Weibull distribution test. **(D)** Box plot of the TTO at the SOC level for caspofungin. Bold bar within the stick: median TTO; Lower end of the stick: 1/4 quantile of the TTO; Upper end of the stick: 3/4 quantile of the TTO. **(E)** The Kaplan-Meier curve shows the cumulative incidence of caspofungin-related ADEs during time. ADE, adverse drug event; SOC, system organ class; PTs, preferred term; q1, 1/4 quantile; q3, 3/4 quantile.

A total of 657 valid TTO reports were analyzed. Most ADEs (591 cases, 89.95%) occurred within the first month of caspofungin administration, with a steady decline in subsequent months (Figures 5A,B). The median time to ADEs onset was 6 days, with an interquartile range (IQR) of 13 days (Q1: 3 days, Q3: 16 days). The upper limit of the 95% confidence interval (CI) for the shape parameter remained below 1 (0.79), indicating an early-failure pattern (Figure 5C). Figure 5D depicts the TTO of caspofungin-related ADEs at the SOC level. The Kruskal–Wallis test indicated significant differences in ADE onset time across the 19 observed SOCs (*P* = 9.0e-08), with rapid onset in renal and urinary disorders (median time, 3 days), psychiatric disorders (4 days), and blood and lymphatic system disorders (4.5 days). In contrast, onset times exceeding 1 week were observed in conditions like skin and subcutaneous tissue disorders (8 days), vascular disorders (8 days), infections and infestations (10 days), and immune system disorders (14 days). More detailed results are available in Supplementary Table S6. Figure 5E presents the Kaplan-Meier plot of cumulative incidence for caspofungin-related ADEs.

### 3.6 Caspofungin-related DILI network construction

**FIGURE 6:**
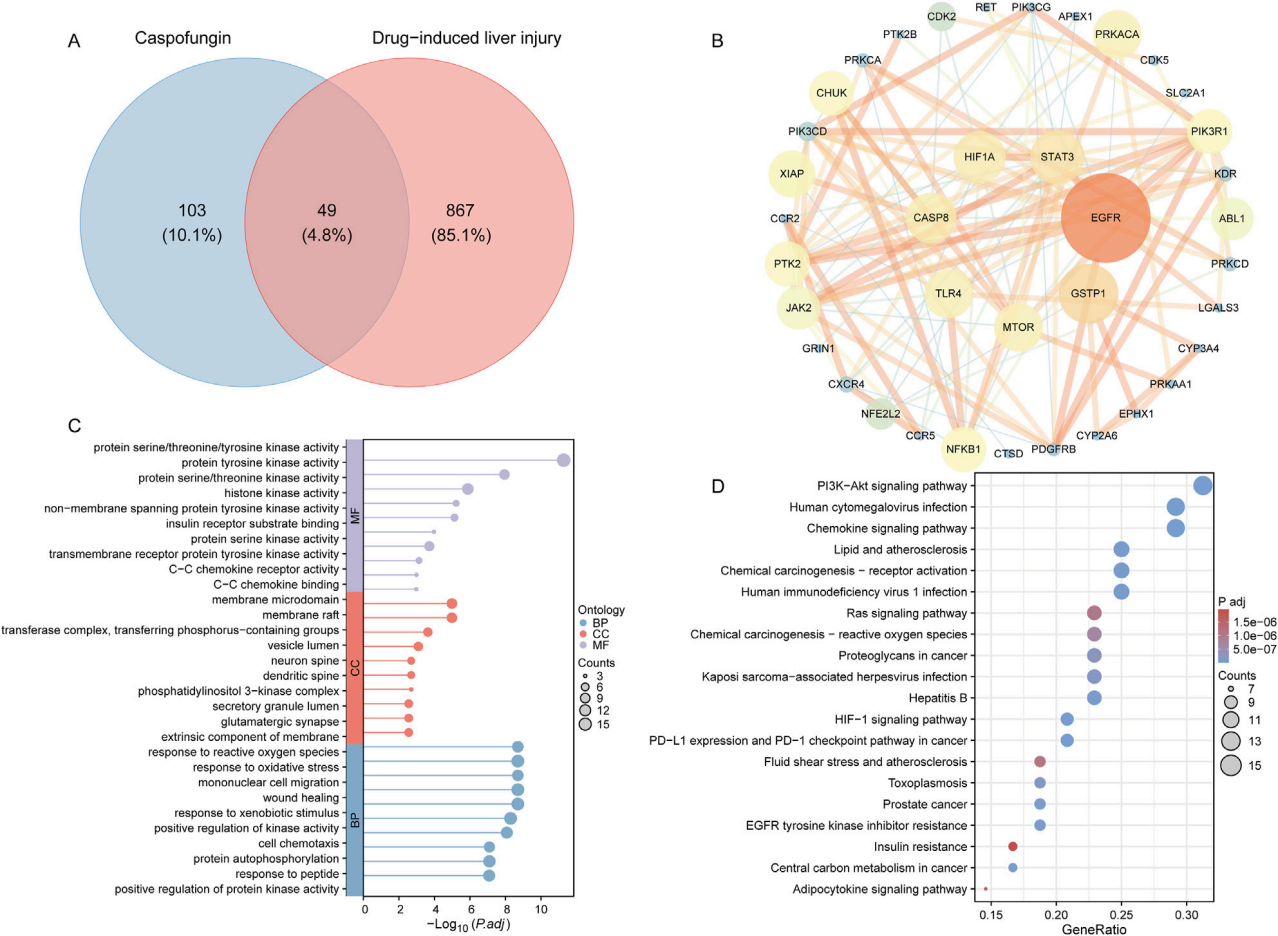
Caspofungin-DILI network construction. **(A)** Intersection target of caspofungin and DILI. **(B)** PPI network construction. Hub genes are positioned in the inner circle, with circle size corresponding to the betweenness centrality score, where larger circles indicate higher scores. **(C)** GO enrichment analysis. Lollipop plots display the top 10 enriched entries for each ontology, with lollipop size representing the count, and the horizontal axis indicating the −log_10_ transformed adjusted *P*-value. **(D)** KEGG enrichment analysis. Bubble plots illustrate the top 20 pathways ranked by enrichment. DILI, drug-induced liver injury; PPI, protein-protein interaction; BP, biological process; CC, cellular component; MF, molecular function; *P*.adj, adjusted *P* value; KEGG, Kyoto Encyclopedia of Genes and Genomes.

In this study, a total of 152 genes associated with caspofungin were identified, comprising 134 drug-related target genes retrieved from the SuperPred database and 22 from the SwissTargetPrediction database. Additionally, 916 genes linked to DILI were identified by combining 882 target genes from GeneCards and 97 from Phenolyzer. The intersection of these two gene sets revealed 49 potential target genes (4.8%) for caspofungin-associated liver injury (Figure 6A). A protein-protein interaction (PPI) network was constructed using the STRING database, applying a high-confidence interaction score cutoff of 0.7. The network included 49 nodes and 122 edges, demonstrating significant PPI enrichment (*P*-value <1.0 × 10^−16^), with core genes including *EGFR*, *GSTP1*, *STAT3*, *HIF1A*, *CASP8*, *TLR4*, and *MTOR* (Figure 6B). GO analysis of the 49 intersecting genes identified 1,324 significantly enriched terms (adjusted *P*-value <0.05). Enriched biological processes were primarily associated with responses to foreign body stimulation, oxidative stress, and wound healing. Cellular components were enriched for membrane rafts and phosphatidylinositol 3-kinase complexes, while molecular functions included kinase activity and chemokine receptor binding (Figure 6C). KEGG pathway analysis revealed 142 significantly enriched pathways. The top five pathways identified were human cytomegalovirus infection, PD-L1 expression and PD-1 checkpoint pathway in cancer, HIF-1 signaling pathway, EGFR tyrosine kinase inhibitor resistance, and the PI3K-Akt signaling pathway (Figure 6D). This analysis offers new insights into the molecular mechanisms of caspofungin-induced DILI and potential therapeutic targets.

## 4 Discussion

### 4.1 Baseline information description

Our analysis of baseline data reveals that, across all three databases, ADEs related to caspofungin are consistently reported more frequently in males than females. In general, females exhibit stronger immune responses than males, leading to higher pathogen clearance rates (Klein and Flanagan, 2016). Specifically, females exhibit higher numbers and activity of innate immune cells, including macrophages and dendritic cells (Villacres et al., 2004), as well as more robust inflammatory immune responses compared to males. Clinical studies have also demonstrated that males tend to have lower CD3^+^ and CD4^+^ T cell counts, reduced CD4^+^/CD8^+^ cell ratios, and weaker Th1 responses than females (Boissier et al., 2003). Similarly, animal studies involving Aspergillus fumigatus and Cryptococcus neoformans infections have shown that female mice exhibit stronger immune responses compared to their male counterparts (Lortholary et al., 2002; Schaefer et al., 2020). Moreover, a review has shown that most fungal diseases have a higher prevalence in males for invasive fungal infections, except for invasive candidiasis (Egger et al., 2022). These immunological and epidemiological lines of evidence strongly support and reinforce our findings. Real-world observations highlight the need for future clinical studies to incorporate sex-specific analyses, which could inform the development of individualized, sex-specific drug use guidelines. However, the absence of weight data in these reports limits the ability to assess its impact on ADEs. Clinical outcomes were concerning, with death reported as the most common outcome across all three databases, likely reflecting the severity of the underlying conditions being treated. Recent studies estimate that invasive fungal infections affect 6.5 million individuals annually and account for 3.8 million deaths, with approximately 68% (2.55 million) directly attributed to fungal diseases (Denning, 2024). The observed differences in the age distribution of ADE reports between CVARD and FAERS may be attributed to several factors, including regional medical practices, drug utilization patterns, and demographic characteristics. In Canada, 59.4% of ADE reports in CVARD involve individuals under the age of 18, a proportion that may reflect the advantages of the Canadian universal healthcare system, which facilitates centralized monitoring and reporting of drug-related adverse events in the pediatric population. In contrast, U.S. commercial health insurance primarily covers the working-age population (18–65 years), with this group being more likely to access expensive antifungal drugs like caspofungin through insurance (Lukowiak et al., 2020). Additionally, FAERS reports are based on voluntary submissions, which may not comprehensively capture all ADEs (Wang et al., 2024). Regional differences in drug prescription practices may also influence the reported proportion of ADEs. A multi-center, randomized, open-label clinical trial involving Canadian pediatric patients with acute myeloid leukemia demonstrated that caspofungin effectively prevents invasive fungal infections (Fisher et al., 2019), a finding that aligns with our observations: the indication antifungal prophylaxis accounted for 56.5% of caspofungin-related ADE reports in CVARD. This suggests that Canadian pediatric patients may be more frequently exposed to caspofungin, with close monitoring of associated adverse events. In contrast, U.S. pediatric patients may use caspofungin more conservatively, leading to relatively fewer reports of adverse events (Lawson and Jick, 1976). Therefore, the age-related differences in SRS ADE reports may be influenced by regional healthcare practices, drug use patterns, age-specific drug safety monitoring, and the methods of reporting in these databases. These demographic and epidemiological characteristics may influence the observed ADE patterns, introducing potential bias into the analysis. Future studies should further analyze regional prescribing habits and reporting behaviors in both databases to gain a deeper understanding of these discrepancies.

### 4.2 Caspofungin-associated SOCs across three databases

Caspofungin, as an antifungal agent, is predominantly associated with SOCs related to general disorders, conditions at the site of administration, and infections, as observed across the FAERS, JADER, and CVARD databases. The high incidence of infections likely reflects the clinical environment in which caspofungin is utilized—primarily among critically ill or immunocompromised patients, who are inherently at higher risk of secondary infections (Lortholary et al., 2011; Pappas et al., 2016; Lortholary et al., 2017). Careful monitoring for secondary infections during caspofungin therapy is essential, as this risk is influenced by the patient’s immunocompromised status and treatment-induced alterations in the microbiome (Dickson, 2016). Strong signals for hepatobiliary disorders across the FAERS, JADER, and CVARD databases highlight the hepatotoxic potential of caspofungin. This underscores the importance of vigilant liver function monitoring during therapy, particularly in patients with pre-existing liver dysfunction or those receiving concomitant hepatotoxic drugs (Nett and Andes, 2016). The robustness of the hepatobiliary signal across multiple databases, particularly the high ROR observed in FAERS (ROR 6.47), reinforces its clinical relevance. These findings underscore the need for proactive measures to identify and address hepatotoxicity, thereby enhancing patient safety during caspofungin therapy.

While shared findings across the FAERS, JADER, and CVARD databases underscore the reproducibility of caspofungin-associated ADE signals, differences in SOC rankings highlight regional variations in patient populations, healthcare settings, and reporting practices. For instance, FAERS captured a broader spectrum of significant SOCs, such as blood and lymphatic system disorders, renal and urinary disorders, and immune system disorders, which were absent in JADER and CVARD (Nomura et al., 2015). This diversity in FAERS likely reflects its larger dataset, population heterogeneity, and comprehensive reporting mechanisms in the United States. The disproportionality analysis revealed strong signals for several SOCs across the databases. Notably, the strongest signal was observed for infections and infestations in CVARD (ROR 14.19), which was significantly higher than the ROR in FAERS (ROR 4.1). This difference may be related to several factors. For example, in Canada (represented by CVARD) caspofungin is frequently used as antifungal prophylaxis in immunosuppressed pediatric patients, such as those with hematological malignancies or undergoing bone marrow transplants (Dvorak et al., 2021; Grimaldi et al., 2025), and therefore the incidence of infection may reflect the immunosuppressed state of the population as well as the increased frequency of antifungal drug use. In addition, in pediatric patients, doses usually need to be adjusted according to body weight and metabolic capacity, which may result in inadequate therapeutic doses, thus affecting the risk of infection and the frequency of reporting. In a recent analysis that included 6,316 children with acute lymphoblastic leukocytes enrolled in the international AIEOP-BFM ALL2009 trial, 68% of infections in the cohort were mycobacteria-related, with significant risk factors for invasive mycosis fungoides being ≥12 years of age and insufficient response to therapy (Lehrnbecher et al., 2023). Alternatively, differences in signal strength between databases may reflect regional variations in medical practice, such as a propensity to report adverse events. These regional differences emphasize the importance of considering local prescribing practices, patient demographics, and healthcare systems when interpreting pharmacovigilance data. Integrating data from multiple pharmacovigilance databases is crucial for achieving a comprehensive understanding of drug safety.

### 4.3 Caspofungin-associated PTs across three databases

Using four independent and complementary analysis methods, multiple positive signals associated with caspofungin were identified, including some unexpected signals. In the FAERS database, signals consistent with the drug label included hypokalemia, respiratory failure, pneumonia, renal failure, and various skin-related ADEs. Hypokalemia was also detected in the JADER database, while renal failure was identified in the CVARD database. Clinical trial data corroborate these findings. A multicenter, double-blind, double-dummy Phase 3 trial comparing the efficacy of rezafungin and caspofungin for treating candidemia and invasive candidiasis reported hypokalemia in 9% of patients, pneumonia in 3%, and acute kidney injury in 8% (Thompson et al., 2023). Similarly, a multicenter, randomized, open-label trial comparing caspofungin with fluconazole for the prevention of invasive fungal disease in children and young adults with acute myeloid leukemia found hypokalemia in 8.7% of patients, alongside six cases of respiratory failure (Fisher et al., 2019). Another double-blind trial comparing caspofungin with amphotericin B deoxycholate for invasive candidiasis reported hypokalemia in 9.9% and nephrotoxicity in 8.4% of patients (Mora-Duarte et al., 2002). Hypokalemia, characterized by a decrease in potassium levels, can significantly disrupt cellular homeostasis by altering the resting membrane potential of cells. This disruption can adversely impact cardiac and respiratory muscles, potentially leading to arrhythmias, cardiac arrest, and respiratory failure. Despite these observations, the exact mechanism by which caspofungin induces hypokalemia remains unknown and warrants further investigation and validation. Previous studies have concluded that echinocandins are generally nontoxic to the kidney (Tragiannidis et al., 2021). However, there have been case reports of severe intravascular hemolysis and secondary renal failure in two patients with hematological disorders after micafungin treatment (Nanri et al., 2009). In our study, renal failure emerged as a positive signal in both the FAERS database (n = 45, EBGM05 = 2.72) and the CVARD database (n = 3, EBGM05 = 2.42). Although no direct cases of caspofungin-associated renal failure have been reported, further investigations are warranted to explore a potential relationship between caspofungin and renal toxicity. Our study also identified several significant skin-related ADEs, including toxic skin eruption, toxic epidermal necrolysis (TEN), Stevens-Johnson syndrome, and dermatitis exfoliative. One case report describes a patient who developed TEN, a severe and life-threatening reaction, after caspofungin administration and subsequently died (Lee et al., 2010). Additionally, another report documents four instances of TEN in patients who developed skin calcification following caspofungin treatment (Colboc et al., 2022). These findings suggest that caspofungin may act as an agonist of the ryanodine receptor (RyR), which is highly expressed in keratinocytes. Activation of RyR agonists induces intracellular calcium flux, disrupting keratinocyte homeostasis. By altering intracellular calcium concentrations, caspofungin may impair the formation of new keratinocytes during early stages of skin healing, potentially leading to recurrent skin calcification and epidermal sloughing. Notably, rare but biologically plausible signals such as photodermatitis were also observed. To our knowledge, there are no reports in the literature on photodermatitis and caspofungin. Although direct evidence is limited, a potential mechanism of action of caspofungin may affect cell structure and function, thereby increasing photosensitivity (Lapoot et al., 2024). In addition, a signal of hepatic damage was present in the same dataset, suggesting that the metabolism or accumulation of reactive intermediates may be altered, which may increase the risk of photosensitization in susceptible populations. Previous report (Hickman et al., 2010) has highlighted the role of hepatic impairment in modulating cutaneous drug reactions, including photosensitive dermatitis, supporting this potential association. These findings suggest that careful monitoring of patients treated with caspofungin for skin-related toxicity and renal function is warranted.

“Drug ineffective” is the most commonly reported significant ADE in the FAERS database. Other prominent signals include product use in unapproved indications, pathogen resistance, and drug resistance. Similar signals were observed in the JADER and CVARD databases, reflecting common challenges in antifungal therapy, particularly for invasive fungal infections characterized by widespread resistance and reduced drug efficacy. Patients with candidemia often have complex underlying medical conditions. The lack of efficacy observed in some cases may be attributed to the severity of these underlying diseases. Additionally, caspofungin is sometimes used off-label in clinical practice, potentially in situations where its efficacy is limited, such as for non-fungal infections or for prophylaxis with insufficient supporting evidence. Emerging fungal pathogens, such as *Candida* auris and related species that exhibit resistance to echinocandins, may also contribute to reduced caspofungin efficacy (Frías-De-León et al., 2020). Resistance to echinocandins like caspofungin is largely mediated by mutations in the FKS genes, which encode subunits of the β-1,3-D-glucan synthase enzyme—the molecular target of the drug (Chowdhary et al., 2018). Specific hot-spot mutations in FKS1 and FKS2, such as in the HS1 region of FKS1, where a serine-to-phenylalanine substitution (S639F), can result in reduced susceptibility to caspofungin, leading to therapeutic failure (Sharma et al., 2022; Izumi et al., 2024; Tian et al., 2024). Moreover, biofilm formation, which provides a protective niche for fungal cells and limits drug penetration, may also contribute to apparent drug ineffectiveness (Lohse et al., 2020). These findings underscore the need for vigilant monitoring of resistance patterns and judicious use of caspofungin to optimize patient outcomes in antifungal therapy. Additionally, drug ineffectiveness may also arise from inappropriate dosages, drug interactions, or subtherapeutic drug concentrations due to rapid drug clearance in critically ill patients (van der Elst et al., 2017). Pharmacokinetic alterations in this population, such as increased renal clearance or hypoalbuminemia, can compromise therapeutic drug levels. These findings highlight the critical importance of confirming microbial infections and performing susceptibility testing before initiating caspofungin therapy. Additionally, healthcare providers must be adequately trained to use caspofungin within evidence-based clinical scenarios to maximize its efficacy and minimize risks.

As mentioned above, secondary infections can significantly impact treatment outcomes. Our analysis of real-world data provides a detailed breakdown of ADEs related to infectious diseases associated with caspofungin, including staphylococcal infection, cytomegalovirus infection, mucormycosis, adenovirus infection, enterococcal infection, and staphylococcal sepsis. These findings offer valuable insights for clinicians to promptly identify and manage these complications. *Staphylococcus aureus* is one of the most prevalent pathogens in the Western world. Studies have shown that caspofungin inhibits the activity of IcaA, reduces extracellular polysaccharide production in *Staphylococcus aureus* biofilms, and enhances the permeability of fluoroquinolones within biofilms, creating a synergistic therapeutic effect (Siala et al., 2016). In addition to infectious ADEs, we identified several serious and unexpected ADEs, including cardio-respiratory arrest, electrocardiogram QT prolongation, and anaphylactic shock. For example, a 14-year-old female patient with acute myeloid leukemia developed a prolonged QT interval, torsades de pointes, and reversible cardiac arrest after transitioning from voriconazole to posaconazole alongside caspofungin therapy (Eiden et al., 2007). Another case involved a 58-year-old female with acute myeloid leukemia who experienced bradycardia and hypotension 45 min after caspofungin infusion, subsequently dying from complete atrioventricular block (Biswal, 2012). While no autopsy was performed to confirm drug-related causality, this event may be linked to hypokalemia or anaphylaxis. An animal study has shown that caspofungin shows relative safety to the heart in mice, but we still need to be vigilant in humans (De Paula et al., 2021). For effective management of serious adverse events such as QT interval prolongation and cardiac arrest, it is recommended that baseline ECG monitoring be performed prior to the administration of caspofungin and that patients’ ECGs be monitored regularly, especially in high-risk groups such as those with cardiac disease, renal insufficiency or electrolyte disorders. In addition, try to avoid combining with other drugs that prolong the QT interval (Li et al., 2023). If drug combinations are necessary, intensive ECG and electrolyte level monitoring should be performed. Anaphylactic shock was also reported in our real-world data. A pooled analysis of the ReSTORE and STRIVE randomized trials identified one case of anaphylactic shock in patients receiving caspofungin (Honoré et al., 2024). Mechanistic studies suggest that caspofungin may activate macrophages *via* Mas-related G protein-coupled receptor X2, triggering mast cell activation and anaphylaxis (Hou et al., 2024). To ensure effective management of caspofungin-induced anaphylaxis, it is recommended to conduct a thorough assessment of the patient’s allergy history and prepare emergency treatment facilities prior to administration, especially in individuals who are first-time users or have a known history of allergies. In summary, our findings provide a comprehensive overview of ADEs associated with caspofungin across multiple SOCs, offering a valuable reference for clinical decision-making. Clinicians should remain vigilant for these new and unexpected signals. If warranted, the FDA should consider updating the drug label and issuing appropriate warnings to enhance patient safety.

### 4.4 TTO analysis

Our analysis demonstrated that the majority of adverse drug events (ADEs) associated with caspofungin occurred within the first 2 months of treatment (n = 640, 97.41%), with the highest frequency observed during the first month (n = 591, 89.95%). Weibull distribution analysis revealed a decreasing likelihood of ADEs over time, as reflected by a median time-to-onset (TTO) of 6 days (IQR: 3–16 days) and an upper limit of the 95% confidence interval for the shape parameter remaining below 1. This supports the observation of a declining probability of ADE occurrence over time. Caspofungin’s limited duration of administration, primarily for infections, may explain why most ADEs occur within the first month. It is important to note that ADEs are often associated with the initiation of drug treatment, potentially leading to biased results. TTO may vary due to several factors, including the pharmacokinetics of caspofungin and its metabolites, as well as underlying pathophysiological mechanisms (Leroy et al., 2014). While there is limited research on the specific timing of ADEs following caspofungin administration, our results showed subgroup differences in the onset of ADEs across SOCs. For example, the shorter TTO for SOCs such as “investigations” may reflect caspofungin’s initial impact on liver metabolism, whereas the longer TTO for “infections and infestations” may be related to delayed immune system responses. These findings suggest the need for targeted management strategies to address SOC-specific ADE timing. Within the first two to 10 days of treatment, efforts should focus on screening for abnormalities in hepatobiliary function and laboratory tests. After 10 days, clinicians should prioritize monitoring infection-related ADEs to prevent serious complications. Such strategies can enhance early detection and improve patient safety during caspofungin therapy.

### 4.5 Bradford Hill causality assessment, molecular targets and signaling pathways in caspofungin-induced liver injury

The results of the Bradford Hill causality assessment suggest a potential association between caspofungin use and DILI, with several criteria supporting this hypothesis. Notably, the strength of the association, consistency across multiple databases, and the temporal relationship between drug administration and the onset of liver injury were observed, which all align with a plausible causal link. According to a study by Zhou et al., based on the FAERS database, the association between echinocandins (including caspofungin) and liver injury was found to be significantly higher compared to triazole antifungals (Zhou et al., 2022). A systematic review and meta-analysis of 39 studies revealed that the incidence of elevated liver enzymes following caspofungin administration was approximately 7% (Wang et al., 2010). Additionally, a retrospective cohort study integrating data from electronic medical records of two large U.S. hospital databases demonstrated that 22.4% of patients in the caspofungin treatment group experienced severe hepatotoxic events within a median observation period of 12 days (Vekeman et al., 2018). Furthermore, an *in vitro* study by Doß et al. found that caspofungin exhibited mild hepatotoxicity. However, in order to better determine the causal relationship between caspofungin and DILI, further validation is needed in future studies. Firstly, *in vitro* experiments using human hepatocyte cell lines could help to elucidate the molecular mechanisms of caspofungin-induced liver injury and potentially identify the key enzymes or pathways involved. In addition, replication of animal models of human liver injury under caspofungin treatment could provide more direct evidence of causality and provide insights into dose-response relationships. Clinical cohort studies of patients treated with caspofungin could provide stronger evidence of the long-term effects of the drug on liver function. By combining data from preclinical and clinical settings, future studies will help clarify whether caspofungin-associated DILI is a true causal phenomenon or simply an association in certain high-risk populations.

This study utilized a network pharmacology approach to identify 49 potential targets associated with caspofungin-induced liver injury. Among these, EGFR, GSTP1, STAT3, HIF1A, CASP8, TLR4, and MTOR emerged as central targets. EGFR, a key regulator of cell proliferation and repair, may play a crucial role in hepatocyte regeneration and repair mechanisms in response to drug-induced damage (Zhang et al., 2018). GSTP1, an antioxidant enzyme that neutralizes reactive oxygen species (ROS), may contribute to the accumulation of oxidative stress when downregulated, thereby triggering apoptosis and liver dysfunction (Zhang et al., 2024). STAT3 activation, commonly linked to inflammatory responses and cell survival signaling, may exacerbate liver damage through excessive inflammatory activity (He and Karin, 2011). Similarly, HIF1A, involved in the HIF-1 signaling pathway, may worsen metabolic disturbances and accelerate non-alcoholic fatty liver disease progression under severe liver stress (Mesarwi et al., 2021). CASP8, a key apoptotic regulator, may directly promote hepatocyte apoptosis when upregulated (Wang et al., 2017). TLR4, critical for recognizing pathogen- and damage-associated molecular patterns, may amplify immune and inflammatory responses, further exacerbating liver injury. Dysregulation of the MTOR signaling pathway may disrupt cell growth, metabolism, and autophagy, leading to hepatocyte dysfunction. Enrichment analysis of these targets provided a comprehensive understanding of the pathways underlying caspofungin hepatotoxicity. Notably, the PI3K/Akt signaling pathway was identified as significantly enriched in caspofungin-related DILI, representing a pivotal finding. Previous studies have shown that AKAP12 deficiency in acute liver damage activates PI3K/Akt phosphorylation, leading to increased PCSK6 expression and the upregulation of downstream inflammation-related genes, which promote macrophage-mediated production of inflammatory factors (Wu et al., 2022). Furthermore, the PI3K/Akt/mTOR pathway is integral to liver regeneration, while the PI3K/Akt1/FOXO1 axis mediates aflatoxin B1-induced hepatotoxicity *via* apoptotic signaling (Ge et al., 2024). These findings suggest that caspofungin may similarly contribute to liver damage by aberrantly activating or inhibiting the PI3K/Akt pathway.

### 4.6 Limitations

Spontaneous Reporting Systems (SRS), including FAERS, JADER, and CVARD, are inherently limited by several factors such as underreporting, missing information, reporting bias, and duplicate submissions (He et al., 2025). Healthcare professionals and patients are more likely to report serious or frequently occurring adverse events, while mild or rare events may be overlooked. This non-systematic and voluntary nature of reporting may lead to underestimation of the true incidence of certain adverse events and restrict the ability to establish reliable causal inferences in the absence of further clinical validation. In addition, some reports may be disproportionately influenced by media coverage, public concern, or legal actions, resulting in “notoriety bias” or temporal reporting bias that further compromises data objectivity (Neha et al., 2021). Moreover, the presence of duplicate reports or incomplete case data may further complicate the accurate assessment of pharmacovigilance signals.

Crucial clinical information—such as concomitant medications, underlying diseases, duration of therapy, drug dosages, and comorbidities—is frequently lacking in these reports, which substantially impedes dose-response analyses and stratified assessments based on patient characteristics (Mao et al., 2024). Consequently, this study did not incorporate dose-related data, and the results cannot be interpreted in the context of drug exposure levels. Further clinically oriented studies are warranted to elucidate the pathogenesis of these adverse events (Xu et al., 2025).

Moreover, heterogeneity in reporting procedures, data structure, and submission thresholds among SRS databases may further exacerbate reporting bias and data variability. The databases used in this study—FAERS (primarily from the United States), JADER (Japan), and CVARD (Canada)—reflect region-specific populations, with differences in patient age, sex distribution, and clinical indications. Such regional biases and disparities in baseline characteristics may limit the generalizability of findings to global or broader populations (Zou et al., 2024).

This study employed disproportionality analysis to detect potential safety signals associated with caspofungin. Although this method is widely used in pharmacovigilance, it only reflects statistical associations between a drug and adverse events and does not establish causality (Wu et al., 2024b). For example, the QT interval prolongation observed in this study may be attributable to hypokalemia, which disrupts potassium ion efflux and delays cardiac repolarization, rather than a direct pharmacological effect of caspofungin.

More importantly, reliance solely on SRS databases for drug safety evaluation may yield misleading conclusions due to the aforementioned biases and data limitations. Therefore, identified safety signals should be interpreted with caution by qualified medical professionals in the context of clinical care, patient variability, and corroborating epidemiological and experimental evidence. In addition, all serious and life-threatening adverse events identified in this study (e.g., hepatotoxicity, hypersensitivity reactions, and arrhythmias) require further validation in real-world clinical settings through independent investigations, including large-scale prospective cohort studies, case-control studies based on electronic health records, and mechanistic studies using animal models and cellular systems to confirm their causality and elucidate underlying biological mechanisms (Chen, 2024).

The results of the Bradford Hill causality assessment suggest a potential association between caspofungin use and DILI, with several criteria supporting this hypothesis. Notably, the strength of the association, consistency across multiple databases, and the temporal relationship between drug administration and the onset of liver injury were observed, which all align with a plausible causal link. An *in vitro* study found that caspofungin exhibited mild hepatotoxicity (Hepatotoxicity of Antimycotics Used for Invasive Fungal Infections: *In Vitro* Results). However, in order to better determine the causal relationship between caspofungin and DILI, further validation is needed in future studies. Firstly, *in vitro* experiments using human hepatocyte cell lines could help to elucidate the molecular mechanisms of caspofungin-induced liver injury and potentially identify the key enzymes or pathways involved. In addition, replication of animal models of human liver injury under caspofungin treatment could provide more direct evidence of causality and provide insights into dose-response relationships. Clinical cohort studies of patients treated with caspofungin could provide stronger evidence of the long-term effects of the drug on liver function. By combining data from preclinical and clinical settings, future studies will help clarify whether caspofungin-associated DILI is a true causal phenomenon or simply an association in certain high-risk populations.

Lastly, the network pharmacology analysis conducted in this study was based on currently available public databases, which may miss emerging drug targets or introduce bias due to incomplete or outdated data sources (Wu et al., 2024a). The predicted drug–gene and signaling pathway interactions, particularly involving PI3K/Akt and HIF-1 pathways, require further experimental validation using *in vitro* systems such as human or animal-derived hepatic cell models to clarify their regulatory roles in caspofungin-associated adverse outcomes.

Despite these limitations, cross-validation across multiple SRS databases in this study has enhanced the robustness of signal detection and facilitated the identification of rare adverse events, thereby providing valuable insight for ongoing pharmacovigilance and safety monitoring of caspofungin.

### 4.7 Future perspectives

#### 4.7.1 Improvement of data quality and reporting protocols

Efforts should be made to enhance the completeness of clinical information in the SRS database. The development of standardized electronic reporting templates could help ensure the inclusion of key clinical data, such as concomitant medication, comorbidities, drug dosage, and treatment duration (de Hoon et al., 2017; Lee et al., 2024). Standardizing reporting procedures and unifying data structures across different SRS databases will also help reduce biases. Furthermore, a regional reporting quality assessment system could be established, with targeted training for healthcare professionals in regions with low reporting rates (Kiguba et al., 2023).

#### 4.7.2 Advanced experimental validation

Results from network pharmacology should be corroborated through experimental validation using *in vitro* models (such as liver cell lines) and animal studies. For instance, exposure experiments using primary human hepatocyte models with dose-time gradient administration of capecitabine could be conducted to assess the phosphorylation levels of key signaling proteins, such as AKT1 and mTOR (Zhao et al., 2021). Additionally, a zebrafish heart toxicity model could be established to dynamically monitor QT interval changes during drug exposure (Coppola et al., 2023).

## 5 Conclusion

Using the FAERS, JADER, and CVARD databases, we identified ADEs associated with caspofungin. Consistent with the drug label, we identified signals included hypokalemia, renal failure, toxic skin eruption, and liver dysfunction, while unexpected signals included prolonged QT interval, septic shock, and cholestasis. Cross-validation across different data sources indicated a strong association between caspofungin and hepatobiliary diseases and infections. Time to onset analysis showed that ADEs were primarily concentrated within the first 2 months of caspofungin treatment. Network pharmacology identified molecular targets and pathways related to caspofungin-induced liver injury, including *EGFR*, *GSTP1*, and the PI3K/Akt signaling pathway, providing deeper insights into its hepatotoxic mechanisms. Despite the limitations of spontaneous reporting databases, these findings underscore the necessity for enhanced safety monitoring and individualized treatment strategies to optimize clinical outcomes.

## Data Availability

The original contributions presented in the study are included in the article/Supplementary Material, further inquiries can be directed to the corresponding authors.
