# Supplementary material for: Unveiling the hidden risk of caspofungin: insights from three adverse event reporting systems and network pharmacology integration
Source: Front Pharmacol. 2025 Aug 13;16:1632488. doi: 10.3389/fphar.2025.1632488 (PMC12380754; doi:10.3389/fphar.2025.1632488)
Supplement: Supplementary file 1 [file Supplementaryfile1.zip › Table 5.DOC]

| **Criteria** | **Description** | **Source/Method** | **Criterion fulfilled** |
| --- | --- | --- | --- |
| Strength of the association | We employed the ROR, PRR, BCPNN, and MGPS to assess the association between caspofungin and DILI. The strength of the disproportionality suggests a robust signal. | Disproportionality analysis | √ |
| Analogy | This association has also been demonstrated with other echinocandin, including micafungin(Valerio et al., 2011; Mullins et al., 2020). | Literature search and labels | √ |
| Biological plausibility/  empirical evidence | The mechanisms by which caspofungin induces DILI remain underexplored. In vitro cell experiments have shown that caspofungin exhibits mild cytotoxicity to hepatocytes at maximum concentrations, manifested as a significant reduction in cell viability(Doß et al., 2017). | Literature search | ? |
| Consistency | Results of disproportionality approaches were consistent across JADER and CVARD. | Disproportionality analysis | √ |
| Specificity | Due to the data limitation in the FAERS database, it is impossible to determine the specificity of the association between caspofungin and DILI. | Disproportionality analysis | ? |
| Temporal relationship | Time-to-onset analysis indicated a temporal relationship between caspofungin treatment and the occurrence of DILI(Marr et al., 2004). | Time-to-onset analysis and literature search | √ |
| Reversibility | This criterion has limited applicability in this context due to the absence of data on rechallenge and dechallenge in the FAERS database. | Not applicable | ? |
| Coherence | Single and multicenter studies(Christopeit et al., 2008; Betts et al., 2009; Jung et al., 2015), literature review(Wang et al., 2010; Kyriakidis et al., 2017), and case report(Marr et al., 2004) support the potential association of DILI with caspofungin. | Literature search | √ |

Notes: √, Relevant Criterium is fulfilled based on pharmacovigilance data. ?, Uncertainty in fulfillment of relevant Criterium.

**Reference：**

Betts R F, Nucci M, Talwar D, Gareca M, Queiroz-Telles F, Bedimo R J, Herbrecht R, Ruiz-Palacios G, Young J A, Baddley J W, Strohmaier K M, Tucker K A, Taylor A F, Kartsonis N A (2009). A Multicenter, double-blind trial of a high-dose caspofungin treatment regimen versus a standard caspofungin treatment regimen for adult patients with invasive candidiasis. Clin Infect Dis, 48(12): 1676-1684

Christopeit M, Eikam M, Behre G (2008). Comedication of caspofungin acetate and cyclosporine A after allogeneic haematopoietic stem cell transplantation leads to negligible hepatotoxicity. Mycoses, 51 Suppl 1: 19-24

Doß S, Potschka H, Doß F, Mitzner S, Sauer M (2017). Hepatotoxicity of Antimycotics Used for Invasive Fungal Infections: In Vitro Results. Biomed Res Int, 2017: 9658018

Jung D S, Tverdek F P, Jiang Y, Kontoyiannis D P (2015). Switching to anidulafungin from caspofungin in cancer patients in the setting of liver dysfunction is associated with improvement of liver function tests. J Antimicrob Chemother, 70(11): 3100-3106

Kyriakidis I, Tragiannidis A, Munchen S, Groll A H (2017). Clinical hepatotoxicity associated with antifungal agents. Expert Opin Drug Saf, 16(2): 149-165

Marr K A, Hachem R, Papanicolaou G, Somani J, Arduino J M, Lipka C J, Ngai A L, Kartsonis N, Chodakewitz J, Sable C (2004). Retrospective study of the hepatic safety profile of patients concomitantly treated with caspofungin and cyclosporin A. Transpl Infect Dis, 6(3): 110-116

Mullins C, Beaulac K, Sylvia L (2020). Drug-Induced Liver Injury (DILI) With Micafungin: The Importance of Causality Assessment. Ann Pharmacother, 54(6): 526-532

Valerio M, Muñoz P, Bouza E (2011). [Liver toxicity of micafungin. Is this drug safe?]. Enferm Infecc Microbiol Clin, 29 Suppl 2: 29-32

Wang J L, Chang C H, Young-Xu Y, Chan K A (2010). Systematic review and meta-analysis of the tolerability and hepatotoxicity of antifungals in empirical and definitive therapy for invasive fungal infection. Antimicrob Agents Chemother, 54(6): 2409-2419
